# Establishing Reference Values for Peripheral Blood Lymphocyte Subsets of Healthy Children in China Using a Single Platform

**DOI:** 10.1155/2022/5603566

**Published:** 2022-08-17

**Authors:** Liangjun Zhang, Huixiu Zhong, Bin Wei, Jiwen Fan, Jingyuan Huang, Yi Li, Weiping Liu

**Affiliations:** ^1^Department of Laboratory Medicine, Zigong First People's Hospital, Sichuan Province, China; ^2^Department of Laboratory Medicine, West China Hospital, Sichuan University, Chengdu, China

## Abstract

Lymphocyte subsets significantly change during childhood; thus, age-matched reference values derived from healthy children are crucial. We established reference values for lymphocyte subsets, including T cells (CD3+), CD4 T cells (CD3 + CD4+), CD8 T cells (CD3 + CD8+), double negative T (DNT) cells (CD3 + CD4-CD8-), B cells (CD3-CD19+), NK cells (CD3-CD56+), and NKT-like cells (CD3 + CD56+) in the peripheral blood of 813 healthy children. We used the method of the international standard document (Clinical Laboratory Standard Institute C28-A3) to establish reference intervals with a single platform. First, we used the Skewness and Kurtosis test to analyze the normality of the data. The nonnormally distributed data was transformed into approximately normal distribution by the Box-Cox transformation. Second, we used the Tukey's method to eliminate outliers. Further, all the subjects were grouped into subgroups according to sex (male and female) and age (0–1 month, 2–12 months, 1–3 years, 4–6 years, and 7–18 years). We used the standard normal deviation test (*Z*-test) to evaluate whether age and sex were possible grouping factors. The analyses indicated age to be an important factor associated with changes in lymphocyte subsets. The absolute number of lymphocyte subsets and total number of lymphocytes, T cells, CD4 T cells, CD8 T cells, and B cells gradually increase from birth to 12 months and then gradually decrease with age. Furthermore, CD4 T cells and the ratio of CD4^+^/CD8^+^ gradually decrease with age. In contrast, CD8 T and DNT cells gradually increase with age. The percentage and number of NK and NKT-like cells gradually increase with age and remain stable between 1 and 18 years of age. In conclusion, the age-related reference intervals established in healthy children in this study can aid in monitoring and assessing the changes in immune levels in diseased conditions.

## 1. Introduction

Lymphocytes are essential components of the human immune system. Immunophenotyping of peripheral blood lymphocyte subsets can provide vital information for diagnosing and treating immunological and hematological disorders [1–3]. The primary cells involved in the adaptive immune response are T and B lymphocytes that dominate the cellular and humoral immunity. The abnormality of the B and T lymphocyte subgroups leads to immune imbalance. T cell subgroup disorder is related to various clinical diseases in children, such as anaphylactic rhinitis, bronchial asthma, cough, and immune thrombocytopenia [4–6]. B cells play a central role in humoral immunity, mainly directed against extracellular infectious pathogens. The most recent international classifications of primary immunodeficiencies (PIDs) and common variable immunodeficiencies (CVID) have highlighted the importance of B cell immunophenotyping and age-specific reference intervals for diagnostic purposes [7].

Lymphocyte reference intervals are lacking for children in China. Foreign diagnostic reagent manuals provide reference intervals for most of the lymphocyte subsets used in clinical laboratories [8]. Some of the previous absolute count reference intervals were established by dual-platform methods that multiply the flow cytometry-derived T-cell percentage by the absolute lymphocyte count derived from hematology analyzers to calculate the absolute count. The use of bead-based single-platform counting methods has significantly reduced interlaboratory variations in absolute cell counts than those with dual-platform methods [9]. The increasing application of single-platform methods for obtaining lymphocyte counts in clinical laboratories requires establishment of reference intervals based on single platform. However, a majority of the previous studies failed to follow the international rules for establishing lymphocyte reference intervals and lack verification. The Clinical Laboratory Standard Institute (CLSI) C28-A3 approved the international standard document to establish medical reference intervals. It provides a method for defining, establishing, and verifying clinical laboratory reference intervals. This study used the method from the CLSI EP28-A3C to establish the relative and absolute numbers of lymphocyte subpopulations in healthy Chinese children from birth to 18 years of age using single-platform method.

## 2. Materials and Methods

### 2.1. Patients

**Figure 1:**
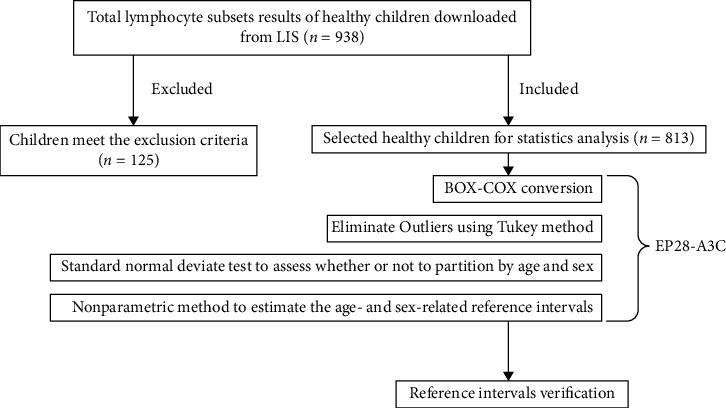
The flow chart of this study for establishing reference values.

We enrolled 938 healthy Chinese children who visited the Zigong First People's Hospital for routine health checkups between 2019 and 2020. We obtained informed consent from parents of minor participants. Exclusion criteria were abnormal blood routine indicators, liver and kidney function indicators, a history of genetic disease and obesity, recent illness, surgery, transfusion, and hospitalization. Finally, 813 cases were included in the study. The study protocol is provided in Figure 1. The study protocol was approved by the Ethics Committee of the Zigong First People's Hospital.

Flow cytometry was used to determine the percentage and number of lymphocytes. The details were as follows: (1) main test reagents and equipment: American Beckman Coulter (BC, California, USA) Multitest Reagent CD45-FITC/CD4-PE/CD8-ECD/CD3-PC5 and CD45-FITC/CD56-PE/CD19-ECD/CD3-PC5, hemolysis, and flow-count fluorospheres, and all antibodies were purchased from BC Company. The American BC Company provided Beckman Coulter NAVIOS flow cytometer instrument. (2) Detection method: the peripheral blood specimens were collected in ethylenediaminetetraacetic acid (EDTA) anticoagulation tubes using reverse sampling, 50 *μ*l of each blood samples was placed in the tubes, 20 *μ*l of multiantibodies was added, mixed well, and incubated at room temperature (20°C-25°C) in the dark for 15 min. Hemolysin (2 ml) was added for 10 min and centrifuged for 5 min with a radius of 5 cm and a rotation speed of 1,500 r/min. The supernatant was removed and washed with 2 ml of acid buffer solution and centrifuged for 5 min with a radius of 5 cm and a rotation speed of 1,500 r/min. The supernatant was removed, and 500 *μ*l of phosphate buffer was added, followed by 50 *μ*l of flow-count fluorospheres. Data on 15,000 cells were acquired on a NAVIOS instrument (BC) and analyzed using Kaluza Analysis Software version 2.1 (BC, California, USA). All experiments were performed in accordance with the relevant guidelines and regulations.

### 2.2. Establishing Reference Values

First, data were analyzed for normality verification by the Skewness and Kurtosis test. If the standard deviation of the corresponding Skewness and Kurtosis was <1.96-times the Skewness and Kurtosis values, these data were judged as nonnormal distribution that were transformed into approximately normal distribution by the Box-Cox transformation. Second, the data outliers were eliminated by the Tukey's method. If the data exceeded the upper and lower limits, they were regarded as outliers. Calculation formula of the upper limit was P75 plus 1.5-times the interquartile range (IQR), and that for lower limit was P25 minus 1.5-times the IQR. Further, all subjects were grouped into subgroups by sex and age. As the distribution was based on the characteristics of children at different stages of growth and development, the age was initially divided into 0–1 month (neonatal period), 2–12 months (infancy stage), 1–3 years (early childhood), 4–6 years (preschool age), and 7–18 years (school age). The standard of age classification was used from a study by Chun [10]. We evaluated whether the grouping was reasonable using the analysis of variance (ANOVA). We performed the standard normal deviation test (*Z*-test) to evaluate the merging of reference intervals. Finally, we calculated the reference range by 95% distribution for the normal distribution and nonparametric data. The above methods were based on the methods in the CLSI EP28-A3C9 document published by the Clinical and Laboratory Standards Institute. Data were analyzed in accordance with the relevant guidelines.

### 2.3. Reference Interval Verification

Forty healthy children in each age group were used for validating the obtained age-adjusted reference intervals. The ratio of individuals outside the reference intervals was calculated; if the ratio was <5%, the reference intervals were verified.

### 2.4. Statistical Analysis

Statistical analyses were performed using Stata 15.0 Statistics (Stata Software Inc., La Jolla, CA, USA) and SPSS Statistics for Windows version 22.0 (SPSS Inc., Chicago, IL, USA). Continuous variables are reported as mean ± standard deviations (SD). The Kolmogorov–Smirnov test was used to assess normality. For nonnormally distributed lymphocyte subsets, comparisons between different age groups were made using nonparametric tests. The Kruskal–Wallis *H* test and *Z*-test were used to evaluate differences between age groups. A *P* < 0.05 was considered to be statistically significant.

## 3. Results

### 3.1. Distribution of Data and Elimination of Outliers

We measured the percentage of lymphocyte subsets, including total T cells (CD3^+^), CD4 T cells (CD3^+^CD4^+^), CD8 T cells (CD3^+^CD8^+^), DNT cells (CD3^+^CD4^−^CD8^−^), B cells (CD3^−^CD19^+^), NK cells (CD3^−^CD56^+^), and NKT-like cells (CD3^+^CD56). Data were analyzed using the Skewness and Kurtosis test; a nonnormal distribution was observed for percentage of almost all cell types, except that of total T cells. Data were transformed into approximately normal distribution using Box-Cox conversion method. The results are shown in Supplementary Table 1. Further, we used the Tukey's method to eliminate some outliers and obtained 813 reference individuals. Detailed parameters before and after elimination are presented in Supplementary Table 2.

### 3.2. Establishment of Reference Interval

A total of 813 healthy children, including 476 boys and 337 girls, participated in the study to establish the lymphocyte subgroup reference interval. We grouped the data by participants' sex. The results of ANOVA and *Z*-test showed that there was no statistically significant difference in lymphocyte reference interval between males and females (*P* > 0.05) and *Z*–values under *z*∗ = 5.9 (*z*∗ = 3(*n*average/120)½ = 3[(*n*1 + *n*2)/240]½). Further, the children were divided into five groups according to their age, as 0–1 month (*n* = 187), 2–12 months (*n* = 154), 1–3 years (*n* = 197), 4–6 years (*n* = 155), and 7–18 years (*n* = 120). The *P* and *Z* values were calculated using ANOVA and *Z*-test, respectively. If the *P* > 0.05 or *P* < 0.05 and *Z* values under *Z*∗, it was not necessary to set certain age by subclass. We combined the reference intervals; if the *P* < 0.05 and *Z* values exceeded *Z*∗, there was statistically significant difference between ages. The results are shown in Supplementary Table 3. The median, 2.5% and 97.5% distribution reference intervals lower and upper limit of lymphocyte subsets after excluding outliers were calculated by the nonparametric method; the results are shown in Table 1.

### 3.3. Changes in Absolute Numbers of Lymphocyte Subsets with Age

**Figure 2:**
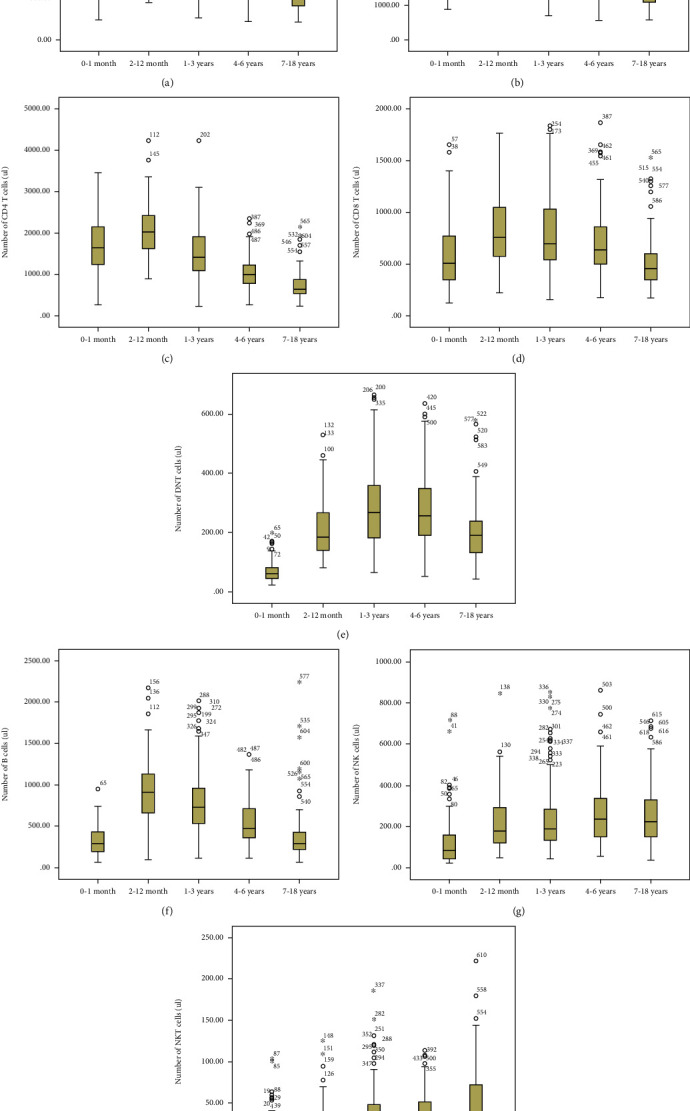
The number of (a) total lymphocyte, (b) T cell, (c) CD4 T cell, (d) CD8 T cell, (e) DNT cell, (f) B cell, (g) NK cell, and (h) NKT cell in Chinese children with different age groups. The uppermost and lowermost lines represent the minimum to maximum. The line through the middle of the box represents the median. The bottom and top of the box represent third quartile and first quartile. Asterisks and circles represent extreme values in the sample data, respectively.

Adaptive immune cells (T and B cells) also changed similarly with age. The numbers of total lymphocytes, T cells, CD4 T cells, CD8 T cells, and B cells gradually increased and peaked at 1 year of age and gradually decreased between 1 and 18 years of age. In contrast, the number of DNT cells gradually increased from birth to 3 years of age and declined from 4 to 18 years of age. The number of NK and NKT-like cells of the innate immunity also peaked around 2–12 months of age but remained stable between 1 and 18 years of age (Figure 2).

### 3.4. Changes in Percentages of Lymphocyte Subsets with Age

**Figure 3:**
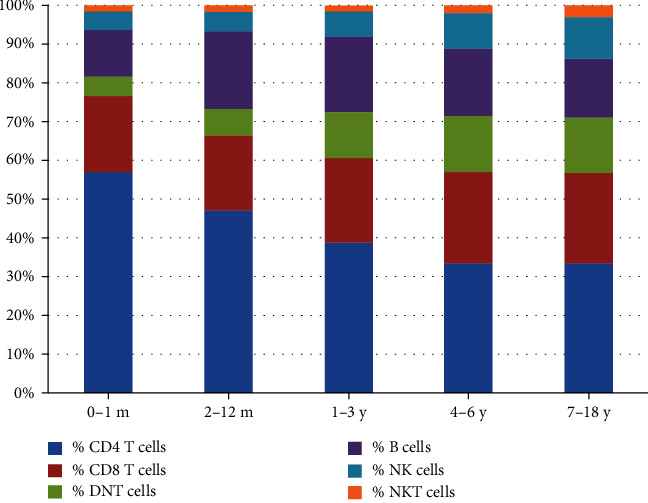
Cell-subset percentages of peripheral blood lymphocytes in Chinese children: distribution by age. Median values of percentages of T cell, CD4 T cells, CD8 T cells cell, DNT cell, B cell, NK cell, and NKT cell.

The total percentage of T cells declined after 1 month of age and remained stable between 2 months and 18 years of age (80.59% to 70.1%). In case of the T cell subgroups, the percentage of CD4 T cells declined slowly with age (56.1% to 34.11%). The percentage of CD8 T cells increased gradually from 18.14% to 22.23%. Interestingly, the percentage of DNT cells increased from 2.78% at 1 month of age to 13.56% at 18 years of age. The percentage of NK and NKT-like cells also showed a similar change, with a gradual increase with age. The percentage of B cells increased from birth (9.84%) to 1 year of age (18.89%) and subsequently slightly reduced (Figure 3).

### 3.5. Verification of Reference Intervals

Some reference individuals were screened from the local reference population for verification (*n* = 40 per group), and their measured values were compared with the original reference values of the reference interval. The factors before and during analysis were consistent with the reference interval in this study. According to the screening criteria, healthy children aged 0–1 month, 2–12 months, 1–3 years, 4–6 years, and 7–18 years were recruited. The percentages of the lymphocyte subgroups from children participating in verification of reference intervals are listed in Table 2. The results showed that all of results were above 95%, all passed verification. The results of reference interval used before (RI^2^) showed that 19% (18/85) were not inside. The reference interval used before (RI^2^) was from foreign diagnostic reagent manuals and other researches (Supplementary Table 4).

## 4. Discussion

The immune system function is imperfect at birth. Genetic inheritance and stimulation of exposed antigens during development gradually mature the immune system [11] that is manifested by the emergence of immune cells and immune molecules from nothing to more in numbers, to naïve, and to mature. Thus, it is essential to establish reference intervals between different ages, especially in children [3]. This is the first study to establish lymphocyte subpopulation reference intervals using the standard way based on the CLSI EP28-A3C regulations in healthy Chinese children using a single platform.

We distributed the percentage and number of lymphocyte subsets of T cells in different age groups. The number of lymphocyte subsets, total lymphocytes, T cells, CD4 T cells, and CD8 T cells increased between 2 and 12 months of age and then gradually decreased. The percentage of lymphocyte subsets, total lymphocytes, Th cells, and CD4^+^/CD8^+^ ratio gradually decreased with age. In contrast, CD8 T cells and DNT cells gradually increased with age. T cells develop in the thymus, colonize the peripheral lymphatic organs after they mature, and circulate in the body. When antigens stimulate the body, T cells participate in the immune response through activation, proliferation, and differentiation stages. T cells are vital in the cellular immune response and also play an important auxiliary role in humoral immune response that is known as the core adaptive immunity. Ding et al. [12] reported similar results that the number of total lymphocytes, T cells, CD4 T cells, CD8 T cells, and B cells is the highest at 6–12 months of age and then gradually decreases with age; also, the proportion of CD4 T cells gradually decreases from birth to 18 years of age, and that of CD8 T cells and DNT gradually increases from birth to 18 years of age. T lymphocytes have cellular innate immune function at birth, and therefore, the immune function of T lymphocytes is predominant. The proportion and number of T lymphocytes are large, and number of T cells gradually increases and peaks at 1 year of age with an increased exposure to external microorganisms and other foreign antigens. The results of the lymphocyte subsets of 106 healthy children in Italy in 2015 [13] showed that the absolute values of T lymphocytes, CD4 T cells, and CD8 T cells gradually decrease with age. In 2011, analyses of lymphocyte subsets in 352 healthy children aged 0–6 years showed that the absolute and relative counts of lymphocyte subsets are the highest in children aged 6–12 months, and the absolute values of CD8 T cells and the CD4^+^/CD8^+^ ratio tend to depend on children's sex [14]. In 2003, analyses of lymphocyte subsets in 807 healthy children from birth to 18 years of age in the United States showed that age is an extremely important factor that affects the percentage of lymphocyte subsets and absolute value distribution [15]. The absolute count of lymphocyte subsets gradually decreases with age, while the absolute counts of lymphocyte subsets are the highest before 1 year of age and gradually decrease with age. These changes between age and cell counts are consistent with those in the present study, although the exact numbers vary with area.

The normal human peripheral lymphoid organs contain a small number of CD3^+^CD4^−^CD8^−^ cells called DNT cells. Recent studies [16] have shown that human DNT cells have an immunosuppressive function and are a new type of immunomodulatory T cells that play an important role during childhood. Peripheral blood DNT cells are primarily derived from the thymus and spleen [4] and to a less extent from thymus-independent pathways, such as activated peripheral blood CD4^+^ and/or CD8^+^ T cells [17]. Priatel et al. [18] showed that T cell receptor (TCR) signal intensity is an important factor in the development of DNT cells. A low intensity of TCR signal can induce generation of mature CD4^+^ T cells, while a high intensity of TCR signal promotes the survival or escape of thymic DNT cells. In the present study, the percentage of DNT cells gradually increased with age, while that of Th cells gradually decreased. This may indicate that CD4 T and DNT cells affect each other during development in children. A few pediatric reference values are available for assessing peripheral blood DNT-cell levels, and findings of the present study provide a useful reference in the evaluation of related diseases.

B cells play a central role in humoral immunity. Most changes in B cell subpopulations occur during childhood, particularly during the first 5 years of life, when children encounter a multitude of antigens. Abnormal production and development of B cells in children can lead to diseases. Children with PIDs and CVIDs often have significantly low levels of CD19 B cells, along with decreased levels of CD3 and CD4 cells [13]. These diseases must be diagnosed by monitoring the number and proportion of B lymphocytes in children. Thus, establishment of normal reference intervals of lymphocyte subsets is imperative for children. The present study suggested that the number of B lymphocytes is the highest between 2 and 12 months of age and then gradually decreases with age. Tosato et al. [19] found that the absolute number of B lymphocytes is high before 2 years of age; it peaks at 2 years of age and then decreases with age. This result is consistent with that of the present study.

The NKT-like cells belong to the innate T lymphocyte subgroup. They express natural killer cell-related receptors and also express TCR that are important for the innate immune system during childhood. In the present study, the percentage and number of NK and NKT-like cells gradually increased with age. In a study by Jia et al. [20], based in north China, the percentage of NK and NKT cells gradually increased with age, while the absolute number of these cells gradually decreased with age. This result is in contrast to the results of the present study; the variability may be attributed to differences in regions and races of the participants.

Age is an important factor in the distribution of lymphocyte subsets. In a majority of studies, the age segmentation of children is very complicated. Each age has a lymphocyte reference value, and there is no effective combination between age groups. This complicates the setting of reference values in practice. Therefore, in the present study, we have approximately classified the reference values according to characteristics of the proportion and the number of lymphocytes. We selected children aged 4–6 years as a group and those aged 0–1 month as a group. Further, we performed ANOVA and *Z*-test to assess the partition reference intervals by subclass. This is the first study to use the standard method to combine age groups with the lymphocyte subset reference intervals in children in China.

A majority of the studies use a dual-platform method to calculate the absolute counts (the percentages of lymphocyte subsets measured by flow cytometer instrument are multiplied by the number of white blood cells (WBC) measured by blood routine instrument). In the present study, a single-platform method was used to calculate the absolute number of lymphocyte subgroups (quantitative microspheres detect the number of lymphoid subsets). This method can directly measure the number of cells, and the results are more objective and accurate, without errors caused by differences in instruments.

The literature [21–27] suggests that lymphocyte subsets change with age and are also affected by various factors, such as ecological environment and social economy. Therefore, there are variabilities between different regions. The lymphocyte reference intervals in the present study were compared with those from studies in Henan [20] and Hong Kong [28] (Table 3). This study used a single-platform, while the two studies used dual-platform methods, and there were also differences in age groupings. The percentage of total T and CD4 T cells in participants in the present study was close to that in children in Hong Kong, but more than that in children in Henan between 2 months and 6 years of age. Moreover, the percentage of CD8 T cells was lower than that in children in Henan and Hong Kong, but the trend was consistent that the percentage increased with age. The absolute numbers of total T cells, CD4 T cells, and CD8 T cells were different between the present and the other two studies. The percentage and absolute numbers of B cells were similar to that in children in Hong Kong, but less than that in children in Henan at 1 year of age. The percentage and absolute numbers of NK cells were significantly lower in the present study than those in the two studies between 2 months and 6 years of age. The differences may be attributed to variability in platforms, the different rates of exposure to antigens after birth, and regional differences in pathogen prevalence.

## 5. Conclusion

The population- and age-specific reference values of lymphocyte subsets established by the present study should aid in appropriate clinical evaluation and treatment of pediatric patients in China, especially as a better understanding of lymphocyte development and maturation, as well as of age-related changes in phenotype, critical for recognition of disease.

## Figures and Tables

**Table 1:** The lymphocyte subset reference interval calculated by nonparametric methods.

Parameter	0-1 month (*N* = 187)	2-12 months (*N* = 154)	1-3 years (*N* = 197)	4-6 years (*N* = 155)	7-18 years (*N* = 120)
% total T cells	80.59 (52.26-89.74)	69.10 (55.87-82.01)	69.10 (55.87-82.01)	69.10 (55.87-82.01)	69.10 (55.87-82.01)
% CD4 T cells	56.06 (28.37-66.26)	47.1 (28.47-63.10)	39.04 (27.15-53.55)	34.11 (22.02-47.62)	34.11 (22.02-47.62)
% CD8 T cells	18.48 (8.96-29.11)	18.48 (8.96-29.11)	21.07 (11.32-31.93)	22.81 (12.88-34.17)	22.81 (12.88-34.17)
CD4+/CD8+ ratio	3.16 (1.29-7.51)	2.52 (1.11-5.47)	1.88 (0.97-3.92)	1.50 (0.79-3.46)	1.50 (0.79-3.46)
% DNT cells	2.78 (0.98-5.16)	5.88 (3.01-23.37)	10.95 (4.31-25.52)	13.55 (5.65-26.44)	13.55 (5.65-26.44)
% B cells	9.84 (3.56-21.33)	18.96 (10.25-31.88)	18.96 (10.25-31.88)	17.00 (8.70-29.21)	14.31 (6.77-30.41)
% NK cells	2.51 (0.76-14.31)	4.12 (1.07-12.23)	5.11 (1.59-20.01)	7.92 (2.33-19.46)	9.96 (1.44-25.51)
% NKT cells	0.64 (0.16-3.20)	0.64 (0.16-3.20)	0.93 (0.23-2.91)	1.22 (0.25-3.65)	2.04 (0.33-6.19)
Number of lymphocytes/*μ*l	3028 (1136-6541)	4498 (2309-7003)	3687 (1615-7096)	2844 (1450-5771)	2043 (1226-4767)
Number of T cells/*μ*l	2333 (1042-5134)	3039 (1612-5230)	2603 (1020-4752)	2018 (998-3975)	1382 (812-3139)
Number of CD4 T cells/*μ*l	1647 (326-3320)	2028 (929-3857)	1410 (484-2809)	996 (420-1974)	638 (373.2-1920)
Number of CD8 T cells/*μ*l	505 (150-1550)	757 (256-1663)	697 (266-1637)	637 (235-1576)	454 (216-1305)
Number of DNT cells/*μ*l	62 (23-170)	185 (86-531)	266 (113-581)	266 (113-581)	190 (66-532)
Number of B cells/*μ*l	292 (85-737)	912 (149-2073)	729 (236-1785)	473 (158-1201)	292 (109-1603)
Number of NK cells/*μ*l	82 (24-665)	210 (60-660)	210 (60-660)	210 (60-660)	210 (60-660)
Number of NKT-like cells/*μ*l	19 (6-94)	35 (9-119)	35 (9-119)	35 (9-119)	35 (9-119)

Values of each population were presented as medians (2.5th to 97.5th) percentile, CD4 T cells (T helper cells), CD8 T cells (cytotoxic T cells), and DNT cells (double-negative T cells).

**Table 2:** The percentages within reference interval of verified population by different reference intervals.

Parameter	0-1 month (*N* = 40)		2-12 months (*N* = 40)		1-3 years (*N* = 40)		4-6 years (*N* = 40)		7-18 years (*N* = 40)	
	Within the RI^1^	Within the RI^2^	Within the RI^1^	Within the RI^2^	Within the RI^1^	Within the RI^2^	Within the RI^1^	Within the RI^2^	Within the RI^1^	Within the RI^2^
% total T cells	97.5%	92.5%	95%	90%	100%	87.5%	97.5%	90%	100%	90%
% CD4 T cells	95%	95%	97.5%	92.5%	100%	90%	95%	92.5%	97.5%	92.5%
% CD8 T cells	97.5%	92.5	95%	92.5%	100%	95%	97.5%	95%	95%	90%
CD4+/CD8+ ratio	97.5%	75%	100%	80%	97.5%	85%	95%	90%	95%	95%
% DNT cells	100%	100%	95%	95%	100%	85%	95%	80%	100%	80%
% B cells	100%	100%	100%	97.5%	95%	92.5%	100%	90%	95%	90%
% NK cells	100%	85%	100%	87.5%	97.5%	85%	100%	90%	97.5%	87.5%
% NKT cells	100%	95%	95%	90%	100%	90%	95%	90%	97.5%	97.5%
Number of lymphocytes/*μ*l	95%	95%	100%	95%	97.5%	80%	95%	85%	95%	85%
Number of T cells/*μ*l	95%	95%	100%	95	95%	80%	100%	85%	95%	85%
Number of CD4 T cells/*μ*l	97.5%	97.5%	100%	97.5%	97.5%	90%	95%	80%	95%	87.5%
Number of CD8 T cells/*μ*l	95%	90%	100%	87.5%	97.5%	85%	95%	90%	97.5%	90%
Number of DNT cells/*μ*l	100%	100%	97.5%	90%	100%	95%	95%	85%	95%	80%
Number of B cells/*μ*l	100%	77.5%	100%	80%	97.5%	82.5%	100%	85%	95%	87.5%
Number of NK cells/*μ*l	95%	90%	100%	90%	97.5%	92.5%	95%	92.5%	95%	90%
Number of NKT cells/*μ*l	97.5%	95%	97.5%	90%	100%	90%	95%	90%	97.5%	92.5%

RI^1^: reference interval defined by this study; RI^2^: reference interval used before; CD4 T cells: T helper cells; CD8 T cells: cytotoxic T cells; DNT cells: double-negative T cells.

**Table 3:** Reference intervals of lymphocyte subsets in children: comparison among different regions in China.

Parameter	Region	2-12 months	1-3 years	4-6 years
% total T cells	Sichuan	69.10 (55.87-82.01)	69.10 (55.87-82.01)	69.10 (55.87-82.01)
	Henan	61 (55-72)	62 (49-72)	65 (54-73)
	Hong Kong	65 (57-73)	69 (66-72)	70 (68-72)
% CD4 T cells	Sichuan	47.1 (28.47-63.10)	39.04 (27.15-53.55)	34.11 (22.02-47.62)
	Henan	36 (28-44)	34 (25-44)	33 (25-42)
	Hong Kong	45 (37-53)	42 (40-44)	34 (32-37)
% CD8 T cells	Sichuan	18.48 (8.96-29.11)	21.07 (11.32-31.93)	22.81 (12.88-34.17)
	Henan	25 (16-34)	24 (17-34)	26 (20-34)
	Hong Kong	25 (22-29)	27 (25-30)	29 (27-31)
CD4+/CD8+ ratio	Sichuan	2.52 (1.11-5.47)	1.88 (0.97-3.92)	1.50 (0.79-3.46)
	Henan	1.46 (0.90-2.37)	1.35 (0.90-2.09)	1.26 (0.84-1.80)
	Hong Kong	1.8 (1.5-2.2)	1.6 (1.4-1.8)	1.3 (1.1-1.4)
% B cells	Sichuan	18.96 (10.25-31.88)	18.96 (10.25-31.88)	17.00 (8.70-29.21)
	Henan	24 (17-33)	21 (13-33)	17 (11-26)
	Hong Kong	17 (10-25)	25 (22-27)	15 (14-16)
% NK cells	Sichuan	4.12 (1.07-12.23)	5.11 (1.59-20.01)	7.92 (2.33-19.46)
	Henan	8 (5-13)	10 (5-20)	12 (7-21)
	Hong Kong	17 (11-24)	7 (6-9)	14 (12-16)
Number of T cells/*μ*l	Sichuan	3039 (1612-5230)	2603 (1020-4752)	2018 (998-3975)
	Henan	3850 (2450-5490)	2960 (1910-4800)	2300 (1150-3530)
	Hong Kong	2500 (1780-3230)	3210 (3260-4360)	2220 (2020-2420)
Number of CD4 T cells/*μ*l	Sichuan	2028 (929-3857)	1410 (484-2809)	996 (420-1974)
	Henan	2240 (1280-3040)	1580 (960-2370)	1180 (800-1730)
	Hong Kong	1720 (1200-2240)	2290 (1950-2620)	1090 (970-1210)
Number of CD8 T cells/*μ*l	Sichuan	757 (256-1663)	697 (266-1637)	637 (235-1576)
	Henan	1530 (840-2380)	1190 (670-1980)	900 (550-1500)
	Hong Kong	970 (700-1240)	1520 (1240-1790)	910 (800-1020)
Number of B cells/*μ*l	Sichuan	912 (149-2073)	729 (236-1785)	473 (158-1201)
	Henan	1600 (850-2390)	910 (580-1830)	670 (400-1060)
	Hong Kong	600 (420-789)	1450 (1180-1720)	1090 (970-1210)
Number of NK cells/*μ*l	Sichuan	210 (60-660)	210 (60-660)	210 (60-660)
	Henan	530 (270-970)	510 (250-1040)	440 (220-810)
	Hong Kong	770 (330-1210)	410 (330-490)	450 (370-530)

## Data Availability

The datasets used and/or analyzed during the current study are available from the corresponding authors on reasonable request.

## Supplementary Materials

Supplementary Materials Lymphocyte subset data normality test and Box-Cox conversion results. Lymphocyte subset data normality test and Box-Cox conversion results. The results of ANOVA analysis and *Z*-test by gender and ages. The data of reference interval determined by the traditional parameter method (RI^2^).
