# Supplementary material for: Establishing Reference Values for Peripheral Blood Lymphocyte Subsets of Healthy Children in China Using a Single Platform
Source: J Immunol Res. 2022 Aug 17;2022:5603566. doi: 10.1155/2022/5603566 (PMC9402384; doi:10.1155/2022/5603566)
Supplement: Supplementary Materials — Lymphocyte subset data normality test and Box-Cox conversion results. Lymphocyte subset data normality test and Box-Cox conversion results. The results of ANOVA analysis and Z-test by gender and ages. The data of reference interval determined by the traditional parameter method (RI2). [file 5603566.f1.doc]

Supplementary Table 1. Lymphocyte subsets data normality test and BOX-COX conversion results

| Parameter | λ | Before Conversion | | | | After Conversion | | | |
| --- | --- | --- | --- | --- | --- | --- | --- | --- | --- |
|  |  | Mean | SD | Skewness | Kurtosis | Mean | SD | Skewness | Kurtosis |
| % total T cells | - | 70.26 | 8.14 | -0.020 | -0.016 | - | - | - | - |
| % CD4 T cells | 0.766 | 40.46 | 10.54 | 0.734 | 0.713 | 20.78 | 4.41 | 0.468 | 1.018 |
| % CD8 T cells | 0.752 | 21.39 | 6.04 | 0.236 | 0.229 | 13.72 | 12.26 | 0.021 | -0.067 |
| CD4+/CD8+ T cell ratio | -0.223 | 2.13 | 1.10 | 1.768 | 4.104 | 0.58 | 0.39 | 0.075 | 0.056 |
| %DNT cells | 0.338 | 11.07 | 7.05 | 3.575 | 39.305 | 3.43 | 1.38 | -0.039 | 0.889 |
| % B cells | 0.687 | 17.07 | 6.38 | 0.373 | -0.050 | 8.61 | 2.66 | 0.021 | -0.067 |
| % NK cells | 0.212 | 7.55 | 5.59 | 1.846 | 5.760 | 2.22 | 1.09 | 0.049 | -0.071 |
| % NKT like cells | 0.004 | 1.28 | 1.05 | 2.088 | 5.992 | -0.03 | 0.748 | 0.070 | -0.423 |
| Number of T cells/μl | 0.232 | 2343.22 | 1014.29 | 1.059 | 1.973 | 21.35 | 2.58 | 0.023 | 0.056 |
| Number of CD4 T cells/μl | 0.343 | 1366.22 | 716.82 | 1.175 | 2.179 | 30.73 | 6.15 | 0.034 | 0.651 |
| Number of CD8 T cells/μl | 0.352 | 707.36 | 364.67 | 1.368 | 3.185 | 24.96 | 5.01 | 0.146 | 0.977 |
| Number of DNTcells/μl | 0.383 | 236.45 | 147.91 | 1.787 | 7.604 | 17.62 | 4.98 | 0.064 | 0.496 |
| Number of B cells/μl | 0.246 | 616.92 | 398.79 | 1.326 | 2.060 | 14.95 | 3.06 | 0.09 | -0.244 |
| Number of NK cells/μl | 0.185 | 243.21 | 189.79 | 2.223 | 8.000 | 8.94 | 2.01 | 0.001 | 0.313 |
| Number of NKT like cells/μl | 0.019 | 39.97 | 28.62 | 1.886 | 5.399 | 3.56 | 0.73 | 0.008 | -0.312 |

CD4 T cells (T helper cells ), CD8 T cells(Cytotoxic T cells ), DNT cells(Double-negative T cells)

Supplementary Table 2. The data before and after outliers were eliminated by the Tukey method.

| Parameter | Before eliminate | | | | | After eliminate | | | | |
| --- | --- | --- | --- | --- | --- | --- | --- | --- | --- | --- |
|  | N | P25 | P75 | Max | Min | N | P25 | P75 | Max | Min |
| % T cells | 813 | 64.92 | 75.05 | 91.99 | 46.76 | 805 | 64.95 | 74.61 | 89.96 | 49.77 |
| % CD4 T cells | 813 | 32.80 | 46.38 | 76.46 | 18.86 | 804 | 32.97 | 45.79 | 67.73 | 18.86 |
| % CD8 T cells | 813 | 17.31 | 25.19 | 42.22 | 7.03 | 806 | 17.71 | 25.23 | 37.70 | 7.03 |
| CD4+/CD8+ T cell ratio | 813 | 1.37 | 2.575 | 7.67 | 0.43 | 811 | 1.39 | 2.44 | 7.67 | 0.67 |
| %DNT cells | 813 | 6.09 | 14.57 | 59.61 | 0.90 | 812 | 6.61 | 14.63 | 33.99 | 0.90 |
| % B cells | 813 | 12.57 | 21.08 | 37.93 | 1.09 | 809 | 13.25 | 21.24 | 34.28 | 3.10 |
| % NK cells | 813 | 3.66 | 9.93 | 43.33 | 0.31 | 809 | 3.755 | 9.83 | 30.68 | 0.59 |
| % NKT like cells | 813 | 0.55 | 1.64 | 7.21 | 0.15 | 813 | 0.55 | 1.645 | 7.21 | 0.15 |
| Number of lymphocytes  total/μl | 813 | 2315 | 4089 | 10846 | 622 | 809 | 2348 | 4076 | 8218 | 869 |
| Number of T cells/μl | 813 | 1612 | 2898 | 7819 | 368 | 807 | 1622 | 2877 | 5906 | 571 |
| Number of CD4 T cells/μl | 813 | 844 | 1793 | 4752 | 222 | 809 | 847 | 1760 | 4234 | 222 |
| Number of CD8 T cells/μl | 813 | 456 | 893 | 2763 | 75 | 808 | 462 | 892 | 1866 | 125 |
| Number of DNTcells/μl | 813 | 138 | 308 | 1268 | 11 | 805 | 150 | 308 | 666 | 23 |
| Number of B cells/μl | 813 | 318 | 820 | 2472 | 26 | 811 | 340 | 824 | 2244 | 67 |
| Number of NK cells/μl | 813 | 123 | 303 | 1603 | 11 | 797 | 128 | 302 | 862 | 23 |
| Number of NKT like cells/μl | 813 | 19 | 52 | 222 | 4 | 812 | 19 | 50 | 222 | 5 |

CD4 T cells (T helper cells ), CD8 T cells(Cytotoxic T cells ), DNT cells(Double-negative T cells)

Supplementary Table 3. The results of ANOVA analysis and Z test by gender and ages

| Parameter | gender | | 0-1month(N=187) | 2-12months(N=154) | 1-3years(N=197) | | 4-6years(N=155) | | 7-18years(N=120) | |
| --- | --- | --- | --- | --- | --- | --- | --- | --- | --- | --- |
|  | P -values | Z -values | P -values | Z -values | P -values | Z -values | P -values | Z -values | P -values | Z -values |
| %total T cells | 0.313 | 1.036 | <0.000 | 8.664 | 0.150 | 2.201 | 0.722 | -0.370 | 0.833 | -0.458 |
| % CD4 T cells | 0.270 | -0.835 | <0.000 | 9.795 | <0.000 | 7.965 | <0.000 | 6.522 | 0.612 | 0.631 |
| % CD8 T cells | 0.061 | 2.368 | 0.434 | -1.420 | 0.015 | -3.543 | <0.000 | -3.720 | 0.945 | 0.013 |
| CD4+/CD8+ T cell ratio | 0.051 | -3.183 | 0.004 | 6.015 | <0.000 | 6.239 | <0.000 | 6.185 | 0.9 | -0.203 |
| %DNT cells | 0.561 | 0.621 | <0.000 | -13.632 | <0.000 | -10.044 | <0.000 | -5.244 | 0.401 | 0.570 |
| % B cells | 0.127 | -1.306 | <0.000 | -16.535 | 0.949 | 0.019 | <0.000 | 4.503 | <0.000 | 3.751 |
| % NK cells | 0.416 | 1.074 | 0.019 | -3.089 | 0.009 | -4.602 | <0.000 | -4.132 | 0.016 | -3.905 |
| % NKT cells | 0.152 | 0.299 | 0.854 | 0.042 | 0.005 | -3.076 | <0.000 | -3.603 | <0.000 | -5.090 |
| Number of lymphocytes  total/μl | 0.421 | 0.802 | <0.000 | -10.612 | 0.001 | 4.968 | <0.000 | 6.991 | <0.000 | 7.062 |
| Number of T cells/μl | 0.543 | 0.788 | <0.000 | -7.021 | <0.000 | 5.420 | <0.000 | 6.673 | <0.000 | 6.902 |
| Number of CD4 T cells/μl | 0.693 | -0.327 | 0.009 | -4.867 | <0.000 | 8.339 | <0.000 | 8.944 | <0.000 | 6.314 |
| Number of CD8 T cells/μl | 0.060 | 2.475 | <0.000 | -7.573 | 0.220 | 1.429 | 0.022 | 2.701 | <0.000 | 5.430 |
|  | 0.230 | 1.343 | <0.000 | -17.148 | <0.000 | -5.946 | 0.860 | 0.112 | <0.000 | 5.922 |
| Number of B cells/μl | 0.733 | -0.915 | <0.000 | -17.075 | 0.050 | 3.526 | <0.000 | 7.264 | <0.000 | 4.383 |
| Number of NK cells/μl | 0.243 | 1.741 | <0.000 | -6.197 | 0.554 | -1.236 | 0.032 | -1.570 | 0.724 | 0.005 |
| Number of NKT cells/μl | 0.094 | 0.415 | 0.001 | -4.991 | 0.260 | -1.735 | 0.545 | -0.304 | 0.108 | -2.725 |

CD4 T cells (T helper cells ), CD8 T cells(Cytotoxic T cells ), DNT cells(Double-negative T cells)

Supplementary Table 4. The data of reference interval determined by the traditional parameter method (RI^2^)

| Parameter | 0-2years | 3-10years | | 11-18years | |
| --- | --- | --- | --- | --- | --- |
| %Total T cells | 39-73 | 56-78 | | 53.6-79 | |
| % CD4 T cells | 25-50 | 27-53 | | 23.8-47.2 | |
| % CD8 T cells | 11-32 | 19-34 | | 11.5-35.1 | |
| CD4+/CD8+ ratio | 0.93-2.5 | | 0.93-2.5 | | 0.93-2.5 |
| %DNT cells | 0-21.9 | | 0-21.9 | | 0-21.9 |
| % B cells | 7-41 | 10-31 | | 6.1-15.8 | |
| % NK cells | 3-16 | 4-25 | | 7.1-24.5 | |
| % NKT cells | 0.9-6.1 | | 0.9-6.1 | | 0.9-6.1 |
| Number of lymphocytes/μl | 1400-8000 | 1100-4200 | | 870-2512 | |
| Number of T cells/μl | 706-2060 | | 706-2060 | | 706-2060 |
| Number of CD4 T cells/μl | 900-5500 | 300-2000 | | 538.4-907.7 | |
| Number of CD8 T cells/μl | 400-2300 | 300-1800 | | 180-694 | |
| Number of DNTcells/μl | 0-339 | | 0-339 | | 0-339 |
| Number of B cells/μl | 600-3100 | 200-1000 | | 83-324 | |
| Number of NK cells/μl | 100-1400 | 90-900 | | 105-564 | |
| Number of NKT cells/μl | 13.6-132.5 | | 13.6-132.5 | | 13.6-132.5 |

CD4 T cells (T helper cells ), CD8 T cells(Cytotoxic T cells ), DNT cells(Double-negative T cells)
